# Ursodeoxycholic Acid Reduces Angiotensin‐Converting Enzyme 2 Activity and Suppresses Cytokine Storm Syndrome in Patients With Coronavirus Disease 2019

**DOI:** 10.1002/mco2.70961

**Published:** 2026-08-30

**Authors:** Zhaowei Tong, Jiawei Zhu, Jianfeng Zhong, Qi Wang, Fuchu Qian, Lili Zhao, Weihong Wang, Yuepao Cai, Kefeng Qin

**Affiliations:** ^1^ Department of Infectious Diseases Huzhou Central Hospital The Affiliated Huzhou Hospital Zhejiang University School of Medicine Huzhou China; ^2^ Department of Laboratory Medicine Huzhou Central Hospital Huzhou China; ^3^ Huzhou Key Laboratory of Precision Medicine Research and Translation for Infectious Diseases Huzhou China; ^4^ Wenzhou University Wenzhou China

**Keywords:** angiotensin‐converting enzyme 2, coronavirus disease 2019, cytokine, cytokine storm syndrome, severe acute respiratory syndrome coronavirus 2, ursodeoxycholic acid

## Abstract

Ursodeoxycholic acid (UDCA) decreases angiotensin‐converting enzyme 2 (ACE2) activities by inhibiting farnesoid X receptor (FXR). To investigate the role of UDCA in decreasing SARS‐CoV‐2 infection and affecting COVID‐19 cytokine levels, COVID‐19 patients (*n* = 142, male = 72, female = 70) were divided into UDCA‐free (*n* = 53) and UDCA (*n* = 89) groups and treated with nirmatasvir/ritonavir or molnupiravir for 5 days. Patients in the UDCA group were additionally administered UDCA for 10 days. On Day 0 (before treatment), 3, 6, and 9 (after anti‐viral drug and/or UDCA treatment), levels of ACE2 in serum and plasma or ACE2 mRNA in blood cells in the UDCA groups were significantly lower than those in the UDCA‐free group. In Calu‐3 cells, UDCA reduced ACE2 protein and mRNA, and blocked the COVID‐19 spike (XBB.1.5) pseudovirus infection. Serum cytokines have been detected in COVID‐19 patients with or without taking UDCA. Among the 46 cytokines analyzed, luminex profiling revealed that 21 proinflammatory cytokines, including CTACK, bFGF, G‐CSF, GM‐CSF, GRO‐α, IL‐1β, IL‐1Ra, IL‐2, IL‐2Rα, IL‐6, IL‐10, IL‐16, IP‐10, MCP‐1, MCP‐3, MIG, TNF‐α, TRAIL, VEGF, IL‐9, and IL‐18, were significantly lower with UDCA treatment (*p* < 0.01), whereas only eotaxin levels increased (*p* < 0.05). Therefore, UDCA reduces ACE2 activity, improves clinical outcomes, and suppresses cytokine storm syndrome (CSS) in COVID‐19 patients.

## Graphical abstract

SARS‐CoV‐2 infection causes COVID‐19 and stimulates immune cells, such as dendritic cells, monocytes, and macrophages, to increase levels of many cytokines. Ursodeoxycholic acid (UDCA) inhibits farnesoid X receptor (FXR), resulting in a decrease in the activity of angiotensin‐converting enzyme 2 (ACE2), the main receptor on the membrane for SARS‐CoV‐2. Therefore, UDCA blocks SARS‐CoV‐2 infection and suppresses COVID‐19 hyper‐inflammatory cytokine responses.

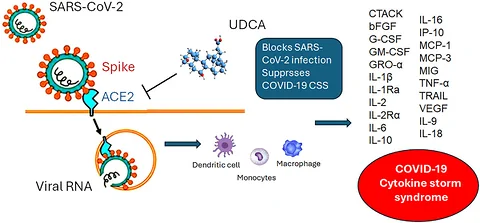

## 1 Introduction

Coronavirus disease 2019 (COVID‐19), caused by severe acute respiratory syndrome coronavirus 2 (SARS‐CoV‐2), has posed an unprecedented global health challenge [1]. SARS‐CoV‐2 is a single‐stranded RNA β‐coronavirus containing structural proteins including spike (S), nucleocapsid (N), membrane (M), and envelope (E) proteins [1]. The virus has evolved into multiple variants, including Alpha, Beta, Gamma, Delta, and Omicron [2, 3]. More recently, the World Health Organization has listed BA.2.86 as a variant of interest and JN.1 as a variant under monitoring [2]. Angiotensin‐converting enzyme 2 (ACE2) serves as the essential receptor on host cell membranes and binds to the spike protein to mediate SARS‐CoV‐2 entry into cells [4], and FXR is a direct regulator of ACE2 at the transcriptional level [5].

The farnesoid X receptor (FXR) is a bile acid‐activated nuclear receptor that functions as a direct transcriptional regulator of ACE2 [5, 6]. Inhibition of FXR by ursodeoxycholic acid (UDCA), an existing generic drug approved for the treatment of cholestatic liver disease, has been shown to reduce ACE2 expression in human lung and liver tissues [5]. UDCA is a hydrophilic bile acid with established safety profiles and anti‐inflammatory properties. By suppressing FXR signaling, UDCA downregulates ACE2 transcription, thereby potentially limiting viral entry into host cells [5]. UDCA has been reported to improve clinical outcomes following COVID‐19 in a retrospective cohort of liver transplant recipients [5].

The clinical utility of UDCA in the context of COVID‐19 remains controversial. Several retrospective studies have reported that UDCA therapy reduces SARS‐CoV‐2 infection risk, alleviates symptoms, and shortens recovery time in patients with chronic liver disease [7, 8]. Conversely, other analyses have concluded that UDCA has no beneficial effect on the prevention of SARS‐CoV‐2 infection in hospitalized patients with liver disease [9, 10, 11]. These conflicting results highlight the need for further investigation into the mechanisms and clinical outcomes associated with UDCA administration in COVID‐19 patients [12, 13, 14, 15, 16, 17].

Beyond viral entry, dysregulated host immune responses contribute significantly to COVID‐19 pathogenesis. Cytokine storm syndrome (CSS), characterized by excessive release of proinflammatory cytokines and chemokines, is a major driver of disease severity and mortality [18, 19]. Elevated levels of interleukin‐6 (IL‐6), tumor necrosis factor‐α (TNF‐α), interferon‐γ‐inducible protein‐10 (IP‐10), and other inflammatory mediators are frequently observed in severe COVID‐19 cases [18, 19, 20]. Therapeutic strategies that simultaneously target viral entry and modulate hyper‐inflammatory responses may offer superior clinical benefits [21, 22].

Based on the premise that UDCA inhibits FXR to reduce epithelial ACE2 expression [5, 6], the aim of this study was to investigate whether UDCA reduces ACE2 expression in COVID‐19 patients and human lung epithelial cells, and to evaluate its effect on the hyper‐inflammatory cytokine response. We demonstrate that UDCA significantly lowers ACE2 protein and mRNA levels in patient blood samples and Calu‐3 cells, blocks SARS‐CoV‐2 spike pseudovirus entry, and suppresses 21 proinflammatory cytokines while increasing eotaxin. These findings provide new clinical and mechanistic evidence that UDCA may serve as an adjunctive therapeutic agent to mitigate cytokine storm syndrome in COVID‐19.

## 2 Results

### 2.1 UDCA Reduces ACE2 Levels in Blood Samples of COVID‐19 Patients

Our primary goal was to observe whether UDCA affects ACE2 activity in COVID‐19 patients (*n* = 142, 72 males and 70 females) in mild, moderate, severe, and critical stages at the Department of Infectious Diseases of Huzhou Central Hospital, from January to May 2023. The patients were randomly divided into the UDCA‐free (*n* = 53) and UDCA (*n* = 89) groups, with body temperatures of 38.6°C ± 1.0°C and 38.9°C ± 0.8°C (*p* = 0.554), respectively, without a significant difference (Table 1). Patients in the UDCA‐free group received nirmatrelvir (300 mg)/ritonavir (100 mg) antiviral therapy twice a day for 5 days (*n* = 45, 85.0%) or molnupiravir (800 mg) twice a day for 5 days (*n* = 8, 15.0%). Patients in the UDCA group received nirmatrelvir (300 mg)/ritonavir (100 mg) twice a day for 5 days (*n* = 78, 87.6%) or molnupiravir (800 mg) twice a day for 5 days (*n* = 11, 12.4%); in addition, UDCA (0.75 g/day) was administered for 10 days.

**TABLE 1:** Patient characteristics.

**Sex, *n* (%)**			
Male	21 (39.6)	51 (57.3)	
Female	32 (60.4)	38 (42.7)	
Age (years)	51.6 ± 20.7	61.8 ± 16.1	0.0013
Range	15–86	26–91	
**COVID‐19 stage, *n* (%)**			
Mild	41 (77.4)	17 (19.1)	
Moderate	3 (5.7)	16 (18.0)	
Severe	8 (15.1)	45 (50.6)	
Critical	1 (1.9)	11 (12.4)	
Temperature before treatment (°C)	38.6 ± 1.1	38.9 ± 0.6	0.0372
Range	36.3–40.0	36.4–40.0	
**Symptoms, *n* (%)**			
Cough	46 (86.8)	77 (86.6)	
Coughing up sputum	5 (9.4)	41 (46.1)	
Sore throat	35 (66.0)	35 (40.7)	
Chest tightness	9 (17.0)	24 (27.0)	
Muscle aches	34 (64.2)	71 (79.8)	
Loss of taste	0 (0.0)	8 (9.0)	
**Basic diseases, *n* (%)**			
Cirrhosis	10 (18.9)	44 (49.4)	
Chronic hepatitis B	32 (60.4)	19 (21.3)	
Hypertension and/or heart disease	2 (3.8)	13 (14.6)	
**Antiviral therapy, *n* (%)**			
Nirmatrelvir/Ritonavir	45 (85.0)	78 (87.6)	
Molnupiravir	8 (15.0)	11 (12.4)	
UDCA (0.75 g/day for 10 days), *n* (%)	0 (0.0)	89 (100.0)	
Time for temperature normalization (days)	2.79 ± 1.81	1.43 ± 0.86	0.0001
Range	1–9	1–6	
Time for respiratory improvement (days)	3.27 ± 1.55	3.24 ± 1.60	0.9131
Range	1–7	1–7	

**FIGURE 1:**
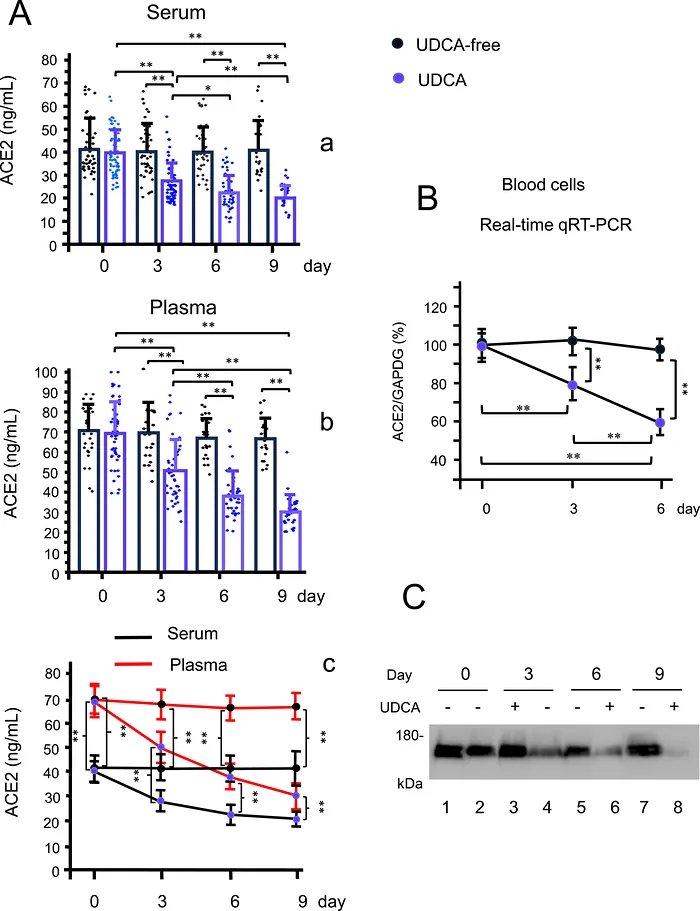
ACE2 activity was decreased in the blood of COVID‐19 patients treated with UDCA. (A) Sandwich ELISA detection of ACE2 concentrations in serum (a) and plasma (b), and comparative analysis of ACE2 levels between serum and plasma over time (c), in UDCA‐free and UDCA groups on Day 0 (before treatment), Day 3, Day 6, and Day 9 (after antiviral and/or UDCA treatment). Individual data points, means, and SD are shown. Serum: UDCA‐free (*n* = 46, 43, 37, 24) and UDCA (*n* = 70, 63, 40, 23) at Days 0, 3, 6, 9. Plasma: UDCA‐free (*n* = 31, 25, 22, 21) and UDCA (*n* = 51, 43, 38, 34) at Days 0, 3, 6, 9. ANOVA with post hoc tests and/or Student's *t*‐test: *p < 0.05, **p < 0.01. (B) Real‐time qRT‐PCR detection of ACE2 mRNA levels in blood cells of COVID‐19 patients in UDCA‐free and UDCA groups on Days 0, 3, and 6. Data are presented as mean ± SD, with individual values (UDCA‐free: *n* = 38, 33, 31; UDCA: *n* = 54, 51, 49). **p < 0.01. (C) Western blot analysis of ACE2 protein in plasma. Representative bands from UDCA‐free (Lanes 1, 3, 5, 7) and UDCA (Lanes 2, 4, 6, 8) groups at Days 0, 3, 6, and 9 are shown.

By detection using Sandwich ELISA, ACE2 levels in serum and plasma of COVID‐19 patients showed significant differences between UDCA‐free and UDCA groups. On Day 0 (before treatment), Days 3, 6, and 9 (after taking antiviral drugs and/or UDCA), ACE2 concentrations in serum and plasma in UDCA‐free group kept approximately 41 ng/mL (*p* = 0.9962) and approximately 68 ng/mL (*p* = 0.6179), but in UDCA group from 40.1 ± 9.6 to 20.8 ± 5.8 ng/mL (*p* = 0.0000) and from 68.8 ± 15.6 to 30.2 ± 7.7 ng/mL (*p* = 0.0000) (Figure 1A; Table 2). Comparison of ACE2 concentrations in serum and in plasma between UDCA‐free and UDCA groups resulted in significant differences on Days 3, 6, and 9 (Figure 1A; Table 2). ACE2 mRNA levels in blood cells of COVID‐19 patients were detected by real‐time qRT‐PCR. On Day 0 (before drug treatment), Day 3 and Day 6 (after taking antiviral drugs and/or UDCA), the levels of ACE2 mRNA in UDCA‐free group were approximately 100.0% (*p* = 0.4133) without significant differences, in UDCA group 100.0% ± 16.3%, 78.2% ± 17.7%, and 58.5% ± 13.2% (*p* = 0.000) with significant differences (Figure 1B; Table 2). The data suggest that (i) ACE2 concentrations in circulation system keep at a constant level in COVID‐19 patients; (ii) ACE2 enzyme activity is significantly reduced in serum and plasma after treatment with UDCA; and (iii) ACE2 levels in plasma are higher than those in serum. Therefore, UDCA reduces ACE2 activity at transcriptional and translational levels in COVID‐19 patients.

**TABLE 2:** Measurement of ACE protein and mRNA levels in blood samples from COVID‐19 patients or Calu‐3 cells by ELISA and real‐time qRT‐PCR.

A. ACE2 concentration in serum and plasma of COVID‐19 patients detected by Sandwich ELISA										
		0		3		6		9		
	Day	ACE2 ng/mL	*n*	ACE2 ng/mL	*n*	ACE2 ng/mL	*n*	ACE2 ng/mL	*n*	*p*
Serum	UDCA‐free	41.2 ± 11.8	46	40.9 ± 12.3	43	41.0 ± 11.0	37	41.6 ± 12.9	24	0.9962
	UDCA	40.1 ± 9.6	70	27.8 ± 8.4	63	23.1 ± 8.4	40	20.8 ± 5.8	23	0.0000
	*p*	0.5965		0.0001		0.0001		0.0001		
Plasma	UDCA‐free	70.6 ± 13.3	31	68.3 ± 12.6	25	66.7 ± 10.1	22	67.0 ± 10.6	21	0.6179
	UDCA	68.8 ± 15.6	51	49.8 ± 16.0	43	38.1 ± 11.9	38	30.2 ± 7.7	34	0.0000
	*p*	0.6205		0.0001		0.0001		0.0001		
Serum	UDCA‐free	41.2 ± 11.8	46	40.9 ± 12.3	43	41.0 ± 11.0	37	41.6 ± 12.9	24	0.9962
Plasma	UDCA‐free	70.6 ± 13.3	31	68.3 ± 12.6	25	66.7 ± 10.1	22	67.0 ± 10.6	21	0.6179
	*p*	0.0001		0.0001		0.0001		0.0001		
Serum	UDCA	40.1 ± 9.6	70	27.8 ± 8.4	63	23.1 ± 8.4	40	20.8 ± 5.8	23	0.0000
Plasma	UDCA	68.8 ± 15.6	51	49.8 ± 16.0	43	38.1 ± 11.9	38	30.2 ± 7.7	34	0.0000
	*p*	0.0001		0.0001		0.0001		0.0001		

B. ACE2 mRNA levels in blood cells of COVID‐19 patients detected by real‐time qRT‐PCR										
Real‐time qRT‐PCR		1		3		6				
ACE mRNA	Day	2‐DDCT	*n*	2‐DDCT	*n*	2‐DDCT	*n*	*p*		
Blood cells	UDCA‐free	100.0% ± 12.7%	38	101.9% ± 15.6%	33	97.4% ± 12.0%	31	0.4133		
	UDCA	100.0% ± 16.3%	54	78.2% ± 17.7%	51	58.5% ± 13.2%	49	0.0001		
	*p*	1.0000		0.0001		0.0001				

C. ACE2 levels in Calu‐3 cells incubated with UDCA detected by Western blots										
Western blots	UDCA µM	0	*n*	25	*n*	100	*n*	200	*n*	*p*
Cula‐3 cell lysates		100.0% ± 4.0%	3	54.3% ± 2.1%	3	53.9% ± 4.5%	3	17.2% ± 9.3%	3	0.0000

D. ACE2 mRNA levels in Calu‐3 cells incubated with UDCA detected by real‐time qRT‐PCR										
ACE mRNA	UDCA µM	0	*n*	25	*n*	100	*n*	200	*n*	*p*
Cula‐3 cell lysates		100.7% ± 4.7%	7	74.6% ± 8.2%	7	65.6% ± 5.0%	7	39.3% ± 7.2%	7	0.0000

*Note*: All data are presented as mean ± SD; analyses by ANOVA and Student's *t*‐test.

### 2.2 UDCA Affects ACE2 in Plasma of COVID‐19 Patients

Western blotting showed ACE2 in plasma (Figure 1C). In the UDCA‐free group, the ACE2 bands on Days 0, 3, 6, and 9 were similar in thickness, but in the UDCA group the band on Days 0, 3, 6, and 9 became thinner. The results suggest that UDCA reduces ACE2 levels in the plasma of COVID‐19 patients.

### 2.3 UDCA Shortens Period of Recovery From Fever in COVID‐19 Patients

The effect of UDCA on clinical outcomes was also observed. In UDCA‐free (*n* = 53) and UDCA groups (*n* = 89), the time of recovery from fever to normal body temperature was 2.79 ± 1.81 and 1.43 ± 0.86 days (*p* = 0.0001), respectively, with a significant difference (Table 1), and the time to respiratory improvement was 3.27 ± 1.55 and 3.24 ± 1.60 days (*p* = 0.9131), respectively, with no significant difference (Table 1), indicating that UDCA improves the clinical outcome of fever returning to normal, but does not affect respiratory symptoms in COVID‐19 patients.

### 2.4 UDCA Reduces the ACE2 Levels in Calu‐3 Cells

**FIGURE 2:**
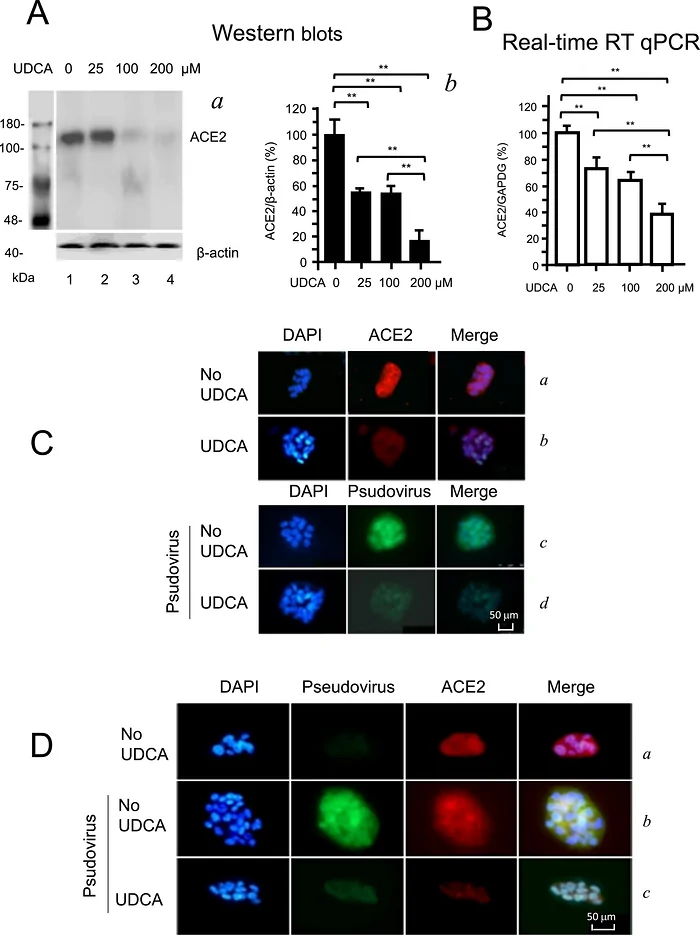
UDCA reduces ACE2 expression and blocks COVID‐19 spike pseudovirus infection in Calu‐3 cells. (A) Calu‐3 cells are incubated with 0, 25, 100, or 200 µM of UDCA for 48 h. (a) Representative Western blot showing ACE2 protein expression in whole‐cell lysates with β‐actin as a loading control. (b) Quantification of ACE2 expression normalized to β‐actin. Data are presented as mean ± SD, with individual biological replicates shown (*n* = 3). ANOVA with post hoc tests: **p < 0.01. (B) ACE2 mRNA levels are detected by real‐time qRT‐PCR and calculated by the 2^−ΔΔCT^ method relative to GAPDH. Data are presented as mean ± SD, with individual biological replicates shown (*n* = 7). ANOVA with post hoc tests: **p < 0.01. (C) Immunofluorescence staining of Calu‐3 cells. (a) ACE2 signal (red) in untreated cells. (b) Diminished ACE2 signal (red) after treatment with 200 µM UDCA for 48 h. (c) COVID‐19 spike (XBB.1.5) pseudovirus infection (green) in the absence of UDCA. (d) Reduced pseudovirus signal (green) in cells co‐incubated with 200 µM UDCA. Scale bars = 50 µm. (D) Merged immunofluorescence images. (a) Uninfected Calu‐3 cells showing ACE2 (red) without pseudovirus signal. (b) Pseudovirus‐infected cells showing both spike (green) and ACE2 (red) signals. (c) UDCA‐treated pseudovirus‐infected cells showing diminished spike (green) and ACE2 (red) signals. Scale bars = 50 µm.

Calu‐3 cells were incubated with 0, 25, 100, and 200 µM of UDCA for 48 h. Western blots showed that ACE2 expression levels in cell lysates were 100.0% ± 4.0%, 54.3% ± 2.1%, 53.9% ± 4.5%, and 17.2% ± 9.3% (*p* = 0.0000), with a significant difference (Figure 2A; Table 2). ACE2 mRNA levels detected by real‐time qRT‐PCR were 100.7% ± 4.7%, 74.6% ± 8.2%, 65.6% ± 5.0%, and 39.3% ± 7.2% (*p* = 0.0000), with significant differences (Figure 2B; Table 2). The results suggest that UDCA reduces ACE2 at the transcription and translational levels in Calu‐3 cells.

### 2.5 UDCA Decreases ACE2 Immunofluorescence Signal and Blocks COVID‐19 Pseudovirus Infection

To observe the effect of UDCA on ACE2 expression in lung cells, Calu‐3 cells were incubated without or with UDCA (200 µM) for 48 h. Immunofluorescence staining showed ACE2 signal (red) in Calu‐3 cells (Figure 2C). After incubation with 200 µM of UDCA, the red ACE2 signal was lower (Figure 2C). COVID‐19 spike (XBB.1.5) protein pseudovirus uses a retroviral vector, in which the envelope protein gene is replaced by the SARS‐CoV‐2 spike protein gene and the green fluorescent protein (GFP) gene. COVID‐19 spike fluorescent signal (green) appeared in pseudovirus‐infected Calu‐3 cells (Figure 2C) and was weaker in pseudovirus‐infected Calu‐3 cells co‐incubated with UDCA (200 µM) for 48 h (Figure 2C). In uninfected Calu‐3 cells, ACE2 signaling (red) was present, but no COVID‐19 spike signal was observed (Figure 2D). After infection with the COVID‐19 spike (XBB.1.5) protein pseudovirus, the spike signal (green) was clearly observed (Figure 2Db). After incubation with UDCA, both the ACE2 signal (red) and the spike signal (green) were much slighter (Figure 2D). The data suggest that (i) UDCA reduces the ACE2 fluorescence signal in Calu‐3 cells; (ii) COVID‐19 spike (XBB.1.5) protein pseudovirus successfully infects Calu‐3 cells with a green fluorescence signal; and (iii) UDCA decreases ACE2 expression, which may block pseudovirus infection.

### 2.6 UDCA Affects Cytokine Levels in COVID‐19 Patients

**FIGURE 3:**
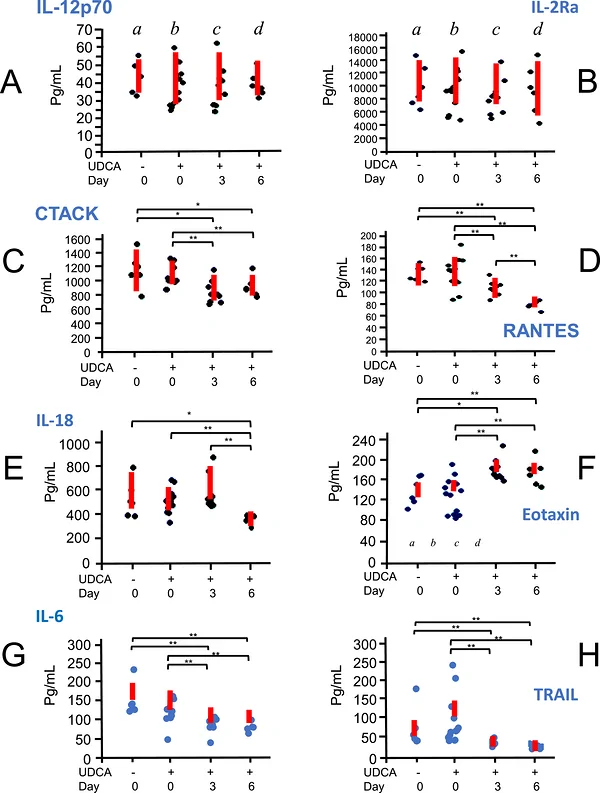
UDCA modulates serum cytokine profiles in COVID‐19 patients. Serum cytokine concentrations are detected using the Bio‐Plex 200 System (Bio‐Rad) in UDCA‐free and UDCA groups on Day 0 (before antiviral and/or UDCA treatment), and Days 3 and 6 (after UDCA treatment). Individual data points, means, and SD are shown. (A and B) Representative cytokines showing no significant response to UDCA: (A) IL‐12p70 and (B) RANTES. (C–H) Representative cytokines showing significant response to UDCA: (C) CTACK and (D) IL‐2Rα are significantly reduced after 3 days of UDCA treatment; (E) IL‐18 is significantly reduced after 6 days of UDCA treatment; (F) eotaxin is significantly increased after 3 days of UDCA treatment; (G) IL‐6 and (H) TRAIL are significantly reduced after 3 days of UDCA treatment. UDCA‐free group: Day 0 (*n* = 53); UDCA group: Day 0 (*n* = 89), Day 3 (*n* = 89), and Day 6 (*n* = 89). ANOVA with post hoc tests and/or Student's *t*‐test: *p < 0.05, **p < 0.01. Complete data for all 46 cytokines are provided in Table 3.

By using the Bio‐Plex 200 System, 46 cytokines in COVID‐19 patients with or without using UDCA were detected. A total of 24 cytokines did not change significantly after using UDCA, including hepatocyte growth factor (HGF), interferon‐alpha 2 (IFN‐α2), interleukin‐3 (IL‐3), interleukin‐5 (IL‐5), interleukin‐7 (IL‐7), interleukin‐8 (IL‐8), interleukin‐12 p70 (IL‐12p70), platelet‐derived growth factor BB (PDGF‐BB), β‐nerve growth factor (β‐NGF), regulated on activation, normal T‐cell expressed and secreted (RANTES), stem cell factor (SCF), stem cell growth factor β (SCGF‐β), tumor necrosis factor β (TNF‐β), interleukin‐13 p40 (IL‐13p40), interleukin‐13 (IL‐13), interleukin‐15 (IL‐15), interleukin‐17 (IL‐17), leukemia inhibitory factor (LIF), macrophage inflammatory protein‐1α (MIP‐1α), macrophage inflammatory protein‐1β (MIP‐1β), macrophage colony‐stimulating factor (M‐CSF), interleukin‐4 (IL‐4), and macrophage migration inhibitory factor (MIF) (Table 3; Figure 3A). UDCA reduced concentrations of 19 cytokines in sera of COVID‐19 patients after taking UDCA for 3 days, including T‐cell‐attracting chemokine (CTACK), basic fibroblast growth factor (bFGF), granulocyte colony‐stimulating factor (G‐CSF), granulocyte‐macrophage colony‐stimulating factor (GM‐CSF), growth‐regulated oncogene‐α (GRO‐α), interleukin‐1 β (IL‐1β), interleukin‐1 receptor antagonist (IL‐1Ra), interleukin‐2 (IL‐2), interleukin‐2 receptor α (IL‐2Rα), interleukin‐6 (IL‐6), interleukin‐10 (IL‐10), interleukin‐16 (IL‐16), interferon‐γ‐inducible protein‐10 (IP‐10), monocyte chemoattractant protein‐1 (MCP‐1), monocyte chemoattractant protein‐3 (MCP‐3), monokine induced by gamma interferon (MIG), tumor necrosis factor‐α (TNF‐α), tumor necrosis factor‐related apoptosis‐inducing ligand (TRAIL), and vascular endothelial growth factor (VEGF) (Table 3; Figure 3B). UDCA reduced concentrations of two cytokines in sera of COVID‐19 patients after taking UDCA for 6 days, including interleukin‐9 (IL‐9) and interleukin‐18 (IL‐18) (Table 3; Figure 3C). UDCA increased the concentration of eotaxin in the sera of COVID‐19 patients after being taken for 3 days (Table 3; Figure 3D). The data suggest that UDCA not only reduces ACE2 expression but also reduces and increases levels of many cytokines (Table S1).

**TABLE 3:** Effects of UDCA on serum cytokine concentrations (pg/mL).

Cytokine	UDCA‐free		UDCA						Significance		Response to UDCA
	Day 0		Day 0		Day 3		Day 6		Day 3	Day 6	
	Mean	SD	Mean	SD	Mean	SD	Mean	SD			
HGF	94.5	27.6	134.1	91.3	133.6	79.9	102.2	33.1			No
IFN‐α2	25.8	2.1	29.4	5.2	27.5	2.5	26.3	2.1			No
IL‐3	28.5	1.3	30.7	9.9	28.3	6.3	25.5	3.6			No
IL‐5	169.5	41.3	140.5	44.7	141.9	43	149.3	31.2			No
IL‐7	22.9	3.7	18.8	4.8	19.4	5.3	18.2	5.3			No
IL‐8	68.9	21.6	77.8	29.7	70.3	20.6	101	30.8			No
IL‐12p70	42.5	8.7	38.8	11.6	36.5	11.8	38	8.6			No
PDGF‐BB	3.7	1.3	3.3	2.2	2.6	1.8	3	1.5			No
β‐NGF	2563.7	1020.5	1743.9	997.1	2469.2	1691.7	2267.9	1890			No
RANTES	10,051.9	2286.6	9293.2	3252	8262.8	2661.5	8250.4	4661.1			No
SCF	177.2	86.1	157.8	55.1	169.1	50.2	151.4	48.1			No
SCGF‐β	77,777.5	7049.1	77,934	12,580	83,957	21,123	94,899	22,831			No
TNF‐β	230.3	20.3	237	33.5	234.8	27.4	213.2	23.3			No
IL‐13p40	22.3	1.8	23.6	3.1	21.7	1.4	22	1.1			No
IL‐13	23.5	2	23.8	4.4	24.1	5.3	26.6	5.3			No
IL‐15	145.5	20.3	125.2	52.5	113.1	44.5	125.8	39.4			No
IL‐17	29.1	5.6	28.4	3.5	28.2	3.6	26.1	4.7			No
LIF	36.5	9.2	33.7	6.1	31	3.7	29	2.1			No
MIP‐1α	59.1	34	106.5	57.7	68.5	77.1	63.1	31			No
MIP‐1β	228	17.4	221.9	28.5	225.6	29.2	207.6	54.5			No
M‐CSF	1791.6	498.7	1824.5	775.5	1932.4	770.9	1957.7	754.6			No
IL‐4	21.7	3.1	19.6	3	19	2.7	18.8	3.2			No
MIF	1791.6	298.7	1824.5	175.4	1932.4	170.9	1957.7	154.6			No
IL‐1α	20.7	1.8	22	3.3	19.3	2.4	19	1.4			No
CTACK	1067.3	217.7	1035.7	137.9	823.6	150.1	817.3	125.3	*	*	Yes
bFGF	29.9	6.1	29	4.2	25	3.1	25	2.7	*	*	Yes
G‐CSF	36.5	8.2	37.4	6.1	28.1	3.8	25.5	3.3	*	*	Yes
GM‐CSF	68.3	10.9	68.2	8.1	53.9	12.9	56.8	5.9	*	*	Yes
GRO‐α	476.1	44.8	455.5	128.6	349.3	94.7	332	60.6	**	**	Yes
IL‐1β	28.4	4.2	29.5	5.5	24.4	2.9	23.7	2.1	*	*	Yes
IL‐1Ra	83.2	9.6	74	15.1	55.7	8.9	29.2	8.2	**	**	Yes
IL‐2	32	3.5	32.7	5.1	28.1	3.6	26	2.1	*	**	Yes
IL‐2Rα	132.7	14.2	136.5	27.1	110.3	12.2	84.2	8.9	**	**	Yes
IL‐6	155	44	148.2	34.6	113.4	26.5	112.8	10.5	*	*	Yes
IL‐10	54	22.7	47	17.3	24.1	2.7	25.5	6.4	**	*	Yes
IL‐16	287.5	41.3	297.7	72.9	194.3	100.8	175	103	*	*	Yes
IP‐10	7862.1	1689.2	4776.6	3724.5	1156.6	1769.2	412.6	483.9	**	**	Yes
MCP‐1	372.7	169.5	257.1	107.8	92.8	45.7	90.5	36.5	**	**	Yes
MCP‐3	45.3	13.2	45.5	13	32.2	2.9	33	2.1	**	*	Yes
MIG	382	56	339	83	97	106	48.5	53	**	**	Yes
TNF‐α	95.6	8.4	95.4	10.1	84.7	10.4	78.9	14.2	*	*	Yes
TRAIL	74.6	14	88.1	16.9	41.4	6.7	35.4	5.6	**	**	Yes
VEGF	399.2	49.9	387.9	132.1	222.6	123.6	202.5	135.2	**	**	Yes
IL‐9	466.8	15.2	474.2	53.8	474.3	39.8	421.2	33.8	ns	*	Yes
IL‐18	523.5	148.6	505.6	95.1	558.2	135.4	372.2	48.3	ns	*	Yes
Eotaxin	139.3	46.5	129.7	33.7	179.1	19.7	174.8	24.8	**	*	Yes

*Note*: Data are presented as mean ± SD.

Abbreviation: ns = not significant.

*p < 0.05, **p < 0.01 vs. ursodeoxycholic acid (UDCA)‐free Day 0 or vs. UDCA Day 0 (as applicable based on post hoc analyses).

## 3 Discussion

Ursodeoxycholic acid (UDCA) prevents SARS‐CoV‐2 infection because UDCA suppresses farnesoid X receptor (FXR), resulting in decreased expression of angiotensin‐converting enzyme 2 (ACE2), the main receptor of SARS‐CoV‐2 [5, 6]. There are mixed reports about the UDCA effect on COVID‐19 clinical outcomes. UDCA therapy reduces COVID‐19 infection risk, alleviates symptoms, and shortens the recovery time in COVID‐19 patients with chronic liver disease [7, 8]. UDCA is associated with a lower hazard and prevents severe COVID‐19 outcomes [14, 15, 16]. Two analyses have concluded that UDCA has no beneficial effect on the prevention of SARS‐CoV‐2 infection in hospitalized patients with liver disease [9, 10, 11]. UDCA serves as an alternative option for preventive drug that protects from SARS‐CoV‐2 infection not only the Delta but also the Omicron variant [17].

In our study, COVID‐19 patients received antiviral therapy with nirmatrelvir/ritonavir or molnupiravir for 5 days and/or UDCA for 10 days. By ELISA measurement, the concentrations of ACE2 in sera and plasma ranged from 10 to 100 ng/mL. ACE2 levels in plasma were higher than those in sera. After COVID‐19 patients started taking UDCA, ACE2 concentrations in sera and plasma were significantly decreased from Day 3 to Day 9. ACE2 mRNA levels detected by real‐time qRT‐PCR were also reduced after UDCA treatment. After taking UDCA, it took less time for COVID‐19 patients to return to normal from fever. We have cultured human lung adenocarcinoma Calu‐3 epithelial cells with different concentrations of UDCA, resulting in UDCA effectively decreasing ACE2 protein and mRNA levels. We have also observed that UDCA reduces ACE2 expression and blocks infection of COVID‐19 spike (XBB.1.5) protein pseudovirus in Calu‐3 cells. Our data provide new evidence that UDCA reduces ACE2 levels in patients with SARS‐CoV‐2 infection and improves COVID‐19 clinical outcomes. We have used human Calu‐3 lung adenocarcinoma cells to demonstrate the UDCA effect on reducing ACE2 levels and blocking COVID‐19 pseudovirus infection. Further experiments are needed to understand the molecular mechanism by which UDCA reduces ACE2 activity.

Cytokine storm syndrome (CSS) is developed in COVID‐19 patients because innate immune cells, such as macrophages, secrete inflammatory cytokines, including proinflammatory cytokines, interleukin‐6 (IL‐6), interleukin‐8 (IL‐8), interleukin‐15 (IL‐15), interleukin‐18 (IL‐18), interleukin‐27 (IL‐27), interferon‐γ (IFNγ), tumor necrosis factor‐α (TNFα), chemokines, growth‐regulated oncogene‐α (GRO‐α), interferon‐γ‐inducible protein‐10 (IP‐10), monokine induced by gamma interferon (MIG), anti‐inflammatory cytokines, interleukin‐1Ra (IL‐1Ra), interleukin‐10 (IL‐10), growth factors, granulocyte colony‐stimulating factor (G‐CSF), and macrophage colony‐stimulating factor (M‐CSF) [18].

Levels of fibroblast growth factor‐2 (FGF‐2), fibroblast growth factor‐basic (FGF‐basic), interleukin‐1β (IL‐1β), interleukin‐7 (IL‐7), IP‐10, macrophage inflammatory protein‐1α (MIP‐1α), macrophage inflammatory protein‐1β (MIP‐1β), and vascular endothelial growth factor (VEGF) are also increased [19, 20, 21, 22]. In addition, concentrations of normal T‐cell expressed and secreted (RANTES), Eotaxin, T‐cell attracting chemokine (CTACK), MIG, hepatocyte growth factor (HGF), stem cell (MIF), leukemia inhibitory factor (LIF), and tumor necrosis factor‐related apoptosis‐inducing ligand (TRAIL) are higher in COVID‐19 patients [18, 20]. MIG, basic‐FGF, and GM‐CSF have been suggested as biomarkers for monitoring COVID‐19 severity [23]. In this study, we have detected 46 cytokines in the serum of COVID‐19 patients with or without administration of UDCA. We have found that UDCA does not significantly change 24 cytokine levels, but significantly decreases or increases 22 cytokine concentrations. SARS‐CoV‐2 infection increases levels of many cytokines, including CTACK, a chemokine that plays a significant role in skin‐related immune responses [24, 25], TRAIL, a death ligand [26], IP‐10, monocyte chemoattractant protein‐1 (MCP‐1) [27], and VEGF [28]. Pregnant women diagnosed with COVID‐19 had higher maternal blood TRAIL and interferon‐γ‐inducible protein‐10 (IP‐10) [29]. In the serum of COVID‐19 patients, there were higher levels of longitudinal cytokines, including inflammatory Th1‐related cytokines IL‐18, IP‐10, MIG, IL‐10, and ARDS‐associated cytokines IL‐6, MCP‐1, IL‐1Ra, IL‐8, GRO‐α, IL‐1Ra, IL‐6, and IL‐8 [30].

Our novel finding is that UDCA decreases levels of cytokines, including CTACK, bFGF, G‐CSF, GM‐CSF, GRO‐α, IL‐1β, IL‐1Ra, IL‐2, IL‐2Rα, IL‐6, IL‐10, IL‐16, IP‐10, MCP‐1, MCP‐3, MIG, TNF‐α, TRAIL, VEGF, IL‐9, and IL‐18. There are a few reports about the response of cytokine levels to antiviral treatment in COVID‐19 patients. Admission of favipiravir or lopinavir/ritonavir for 7 days does not change the high serum levels of cytokines, including IL‐6, IL‐8, and IFNγ [31]. After treatment with remdesivir for 5 days, serum levels of IL‐6, TNF‐α, and IFN‐γ were reduced, whereas serum levels of IL‐4 were increased [32]. Bacille Calmette–Guérin vaccination reduces three cytokine responses, including CTACK, TRAIL, and VEGF [33, 34]. In this study, the only serum cytokine increased after taking UDCA is eotaxin, a chemokine that plays a crucial role in the recruitment and activation of eosinophils. Therefore, an important role of UDCA is the adjustment of COVID‐19 cytokine symptoms, mostly suppressing SARS‐CoV‐2‐induced cytokine levels.

### 3.1 Limitations

This study has several limitations. First, the sample size was relatively modest, and the patients were enrolled from a single center, which may limit the generalizability of the findings. Second, the observation period was relatively short; long‐term follow‐up data on patient recovery and potential recurrence are lacking. Third, while we demonstrated that UDCA reduces ACE2 expression and blocks pseudovirus infection in vitro, the detailed molecular mechanism by which UDCA modulates individual cytokine signaling pathways remains to be fully elucidated. Fourth, the cytokine analysis was performed on serum samples, and tissue‐specific cytokine profiles were not assessed. Finally, this was not a randomized controlled trial; although patients were randomly grouped, potential confounding factors such as baseline liver disease status may have influenced outcomes. Larger, multicenter randomized controlled trials are warranted to validate these findings.

## 4 Conclusions

We have compared ACE2 activity in COVID‐19 patients treated with or without UDCA and found that UDCA reduces ACE2 levels in serum and plasma, and ACE2 mRNA in blood cells, which may lead to a shorter time to return to normal body temperature. UDCA can also reduce ACE2 levels and block COVID‐19 spike pseudovirus infection in Calu‐3 cells. A novel finding is that UDCA decreases 21 cytokine levels in sera of COVID‐19 patients, demonstrating that UDCA might be a potential agent to suppress cytokine storm syndrome caused by SARS‐CoV‐2 infection.

## 5 Materials and Methods

### 5.1 Patients

COVID‐19 patients (*n* = 142, 72 males and 70 females) were evaluated for mild, moderate, severe, and critical stages at the Department of Infectious Diseases of Huzhou Central Hospital, from January to May 2023. The patients were randomly divided into the UDCA‐free (*n* = 53) and UDCA (*n* = 89) groups, with body temperatures of 38.6°C ±1.0°C and 38.9°C ± 0.8°C (*p* = 0.554), without a significant difference (Table 1).

### 5.2 Treatment

Patients in the UDCA‐free group (*n* = 53) received nematevir (300 mg)/ritonavir (100 mg) antiviral therapy twice a day for 5 days (*n* = 45, 85.0%) or monoravir (800 mg) twice a day for 5 days (*n* = 8, 15.0%). Patients in the UDCA group (*n* = 89) received nirmatavir (300 mg)/ritonavir (100 mg) twice a day for 5 days (*n* = 78, 87.6%) or molnupiravir (800 mg) twice a day for 5 days (*n* = 11, 12.4%), in addition to UDCA (0.75 g/day) for 10 days.

### 5.3 Detection of ACE2 Levels in Human Blood Samples

Sera, plasma, and blood cells were collected from assessed COVID‐19 patients on Day 0 (pre‐treatment), Days 3, 6, and 9 (post‐treatment) according to the standard protocols. Sandwich ELISA was performed using a 96‐well ELISA plate according to the standard protocol. Reagents included the capture antibody, rabbit anti‐ACE2 monoclonal antibody (mAb) (Abcam); ACE2 protein (Abcam) as a standard antigen; serum or plasma as antigens for detection; primary antibody, mouse anti‐ACE2 mAb (Abcam); secondary antibody, HRP‐labeled goat anti‐mouse IgG. A standard curve of ACE2 concentrations was calculated to determine ACE2 concentration in each sample. Western blots with mouse anti‐ACE2 mAb (Abcam) as the primary antibody were used to detect ACE2 bands in plasma.

### 5.4 Cell Culture

Calu‐3 epithelial cells (human lung adenocarcinoma cells) (ATCC HTB‐55) were cultured in MEM medium containing 10% fetal bovine serum (FBS), 100 IU/mL penicillin, and 100 µg/mL streptomycin at 37°C and 5% CO_2_. Cells were spread and cultured in antibiotic‐free medium in 10‐cm plates for 24 h until the cells reached approximately 80% confluence, followed by incubation of UDCA at 0, 20, 100, and 200 µM for 48 h. Cell lysates were analyzed by Western blots for ACE2 expression levels.

### 5.5 Detection of ACE2 mRNA Levels by Real‐Time qRT‐PCR

The Total RNA Extraction Kit (Solarbio Life Science, Beijing) was used to extract the total RNA from whole blood cells of COVID‐19 patients in UDCA‐free and UDCA groups or Calu‐3 cells. PCR primers were as follows: ACE2: forward 5'‐CCA CTG CTC AAC TAC TTT GAG CC‐3' and reverse 5'‐CTT ATC CTC ACT TTG ATG CT‐3'; GAPDH: forward 5'‐GAA GGT GAA GGT CGG AGT C‐3' and reverse 5'‐GAA GAT GGT GAT GGG ATT TC‐3' [35]. The total RNA of each sample was reverse transcribed into complementary DNA by Perfect Real Time Kit (Takara Bio Inc., Japan). The real‐time quantitative PCR (qPCR) was performed with TB Green Permix Ex TagII Kit (Takara) in ABI QuantStudio 5 (Thermofisher). The target gene expression was calculated using the 2^−ΔΔCT^ method.

### 5.6 Pseudovirus Infection and Immunofluorescence Staining

Calu‐3 cells were placed on 1.4‐cm coverslips in the well of a 24‐well plate and incubated for 24 h. After washing with phosphate‐buffered saline (PBS), cells were incubated in 0.2 mL medium without or with 0.2 µL of COVID‐19 spike (XBB.1.5) protein pseudovirus (Yeasen Biotechnology, Shanghai Co. Ltd) for 6 h. After removing the infection medium, cells were incubated in 0.2 mL medium without or with UDCA (200 µM) for 24 h. Cells were fixed with 4% paraformaldehyde in PBS and permeabilized with 0.1% Triton X‐100 in PBS. After blocking with 1% bovine serum albumin (BSA) in PBS, cells were incubated with rabbit anti‐ACE2 mAb (Abcam) and then incubated with Alexa Fluor 488 F(ab')‐goat anti‐rabbit IgG (H+L) (Invitrogen). The stained cells were dried in air and then mounted with Vectashield mounting medium for fluorescence (H‐100 containing 4'‐6‐diamidino‐2‐phenylindole (DAPI) (Vector Laboratories Inc., Burlingame, CA, USA). The cell morphology and immunofluorescence were observed and captured using a Leica fluorescence microscope (Wetzlar, Germany).

### 5.7 Detection of Cytokine Levels in Sera of COVID‐19 Patients

The Bio‐Plex 200 System (Bio‐Rad) was used to detect levels of 46 cytokines. Sera from COVID‐19 patients with or without taking UDCA, blank and standard samples (50 µL) were incubated with washed beads (50 µL) in a 96‐well assay plate pasted with an aluminum film at 850 rpm at room temperature for 30 min. After the incubation, the liquid in each well was removed and then washed with wash buffer (100 µL) three times. The diluted antibodies for detection (25 µL) were added to the well and then incubated at 850 rpm at room temperature for 30 min, pasted with a new aluminum film. After the incubation, the liquid in each well was removed and then washed with wash buffer (100 µL) three times. The detection antibody (50 µL) was added into each well and then incubated at 850 rpm at room temperature for 30 min pasted with a new aluminum film. After the incubation, the liquid in each well was removed and then washed with wash buffer (100 µL) three times. The liquid contained in each well was removed and then washed with wash buffer (100 µL) three times. The beads were resuspended in assay buffer (125 µL) in each well and then incubated at 850 rpm at room temperature, pasted with a new aluminum film. At the end of the day, the aluminum film was removed, and the plate was read and calculated with Bio‐Plex Pro Human Cytokine Screening 48‐Plex Panel (Bio‐Rad).

### 5.8 Statistical Analysis

ANOVA was applied to define differences between treatment groups, followed by post hoc multiple comparison tests to define specific group differences (Statpages). Student's *t*‐test was applied to assess the relationship between different variables (GraphPad, Prism). The value of *p* < 0.05 is considered significant, and *p* < 0.01 very significant.

## Author Contributions

Z.T.: conceptualization, investigation, data curation, writing – original draft, and project administration. J.Z.: methodology, formal analysis, and resources. Q.W.: methodology, sample collection, formal analysis, and investigation. F.Q.: validation, formal analysis, and investigation. L.Z.: experiment operation and data analysis. W.W.: supervision, project administration, and funding acquisition. K.Q.: methodology, formal analysis, supervision, project administration, writing and revising the manuscript, and funding acquisition. All authors have read and agreed to the published version of the manuscript.

## Funding

This work was supported by a grant from Huzhou Public Welfare Research Project (grant number: 2024GZB10).

## Ethics Statement

The patient part of the study conformed to the Declaration of Helsinki (1964), and the protocol was approved by the Ethics Committee of Huzhou Central Hospital (Ethics No: 2023001‐02). All human subjects provided written informed consent.

## Conflicts of Interest

The authors declare no conflicts of interest.

## Supporting information




**Supporting File S1**: mco270961‐sup‐0001‐tableS1.docx

## Data Availability

All data and materials supporting the findings of this study are available within the paper.
