# Supplementary material for: Ursodeoxycholic Acid Reduces Angiotensin‐Converting Enzyme 2 Activity and Suppresses Cytokine Storm Syndrome in Patients With Coronavirus Disease 2019
Source: MedComm (2020). 2026 Aug 30;7(9):e70961. doi: 10.1002/mco2.70961 (PMC13527531; doi:10.1002/mco2.70961)
Supplement: Supplementary file 1 [file MCO2-7-e70961-s001.docx]

**Ursodeoxycholic acid reduces Angiotensin-converting enzyme 2 expression and attenuates hyper-inflammatory cytokine responses in Coronavirus disease 2019**

Zhaowei Tong^1,2,3^, Jiawei Zhu^4^, Jianfeng Zhong^1,3^, Qi Wang^1,3^, Fuchu Qian^2,^, Lili Zhao^2^, Weihong Wang^1,3^, Zhengyi Bao^1,2,3^, Yuepiao Cai^4*^, Kefeng Qin^1,2,3^*

1. Department of Infectious Diseases, Huzhou Central Hospital, The Affiliated Huzhou Hospital, Zhejiang University School of Medicine, Huzhou, China

2. Department of Laboratory Medicine, Huzhou Central Hospital, Huzhou, China.

3. Huzhou Key Laboratory of Precision Medicine Research and Translation for Infectious Diseases, Huzhou, China

4. School of Pharmaceutical Sciences, Wenzhou Medical University, Wenzhou, China

Running title: UDCA reduces ACE2 and affects cytokine levels in COVID-19

* Correspondence:

Kefeng Qin

**Supplementary Table 1** Serum cytokine concentrations related wit COVID-19

A. Five cytokine levels in a reference [50] and this study

| Data from Reference (Zhang et, 2020) | | | | | UDCA free |  | UDCA | | |
| --- | --- | --- | --- | --- | --- | --- | --- | --- | --- |
| Cytokine | Healthy | Mild | Moderate | Severe/ | Day 0 |  | Day 0 | Day 3 | Day 6 |
|  |  |  |  | Critical |  |  |  |  |  |
| IL-6 | ≈ 1 | 5–10 | 20–50 | 100–500 | 155±44 |  | 148.2±34.6 | 113.4±26.5 | 112.8±10.5 |
| IL-1Ra | ≈ 10 | 50 | 200 | 400–800 | 83.2±9.6 |  | 74.0±15.1 | 55.7±8.9 | 29.2±8.2 |
| IL-8 | ≈ 5 | 15 | 30 | 60–120 | 68.9±21.6 |  | 77.8±29.7 | 70.3±20.6 | 101.0±30.8 |
| IP-10 | ≈ 50 | 200 | 800 | 2000–5000 | 7862.1±1689.2 |  | 4776.6±3724.5 | 1156.6±1769.2 | 412.6±483.9 |
| IL-10 | ≈ 2 | 5 | 10 | 20–40 | 54±22.7 |  | 47±17.3 | 24.1±2.7 | 25.5±6.4 |

B. Six cytokine levels in a reference [51] and this study

| Data from reference (Ozger et al, 2021) | | | UDCA free |  | UDCA (mean±SD) | | |
| --- | --- | --- | --- | --- | --- | --- | --- |
| Cytokine | Healthy donor | Median (IQR) in moderate | Day 0 (mean±SD |  | Day 0 | Day 3 | Day 6 |
| IL-6 | 1.3 (0.9–2.1) | 18 (10–31) | 155±44 |  | 148.2±34.6 | 113.4±26.5 | 112.8±10.5 |
| IL-1β | 0.7 (0.3–1.1) | 3.3 (1.8–9.6) | 28.4±4.2 |  | 29.5±5.5 | 24.4±2.9 | 23.7±2.1 |
| TNF-α | 6.2 (4.1–8.5) | 14 (9–20) | 230.3±20.3 |  | 237±33.5 | 234.8±27.4 | 213.2±23.3 |
| IP-10 | 62 (45–89) | 610 (174–5 594) | 7862.1±1689.2 |  | 4776.6±3724.5 | 1156.6±1769.2 | 412.6±483.9 |
| MCP-1 | 75 (58–98) | 1 350 (765–2 297) | 372.7±169.5 |  | 257.1±107.8 | 92.8±45.7 | 90.5±36.5 |
| VEGF-A | 115 (78–152) | 362 (153–658) | 399.2±49.9 |  | 387.9±132.1 | 222.6±123.6 | 202.5±135. |

Note: concentration (pg/mL)
